# Dietary n-3-polyunsaturated fatty acids and energy balance in overweight or moderately obese men and women: a randomized controlled trial

**DOI:** 10.1186/1743-7075-6-24

**Published:** 2009-05-29

**Authors:** Mario Kratz, Holly S Callahan, Pamela Y Yang, Colleen C Matthys, David S Weigle

**Affiliations:** 1 Department of Medicine, Division of Metabolism, Endocrinology & Nutrition, University of Washington, 1959 NE Pacific Street, Seattle, Washington 98195, USA; 2 Fred Hutchinson Cancer Research Center, Cancer Prevention Program, 1100 Fairview Ave N, Mail stop M4-B402, Seattle, Washington 98109, USA; 3 General Clinical Research Center at the University of Washington, 1959 NE Pacific Street, Seattle, Washington 98195, USA

## Abstract

**Background:**

Dietary n-3-polyunsaturated fatty acids (n-3-PUFA) have been shown to reduce body weight and fat mass in rodents as well as in humans in one small short-term study. We conducted this controlled randomized dietary trial to test the hypothesis that n-3-PUFA lower body weight and fat mass by reducing appetite and *ad libitum *food intake and/or by increasing energy expenditure.

**Methods:**

Twenty-six overweight or moderately obese (body mass index 28–33 kg/m^2^) men and women were included, and received either a diet rich in n-3-PUFA from both plant and marine sources or a control diet. Diets were administered in an isocaloric fashion for 2 weeks followed by 12 weeks of *ad libitum *intake. The n-3-PUFA and control diets were identical in all regards except for the fatty acid composition. All foods were provided to subjects, and leftovers were weighed back to assess actual food intake accurately for each day of the study. This design gave us 80% power to detect a difference in weight change between the n-3-PUFA and control diet groups of 2.25 kg at an α-error level of 5%.

**Results:**

Both groups lost similar amounts of weight when these diets were consumed *ad libitum *for 12 weeks [mean (SD): -3.5 (3.7) kg in the control group vs. -2.8 (3.7) kg in the n-3-PUFA group, F_(1,24) _= 13.425, p = 0.001 for time effect; F_(1,24) _= 0.385, p = 0.541 for time × group interaction]. Consistent with this finding, we also found no differences between the n-3-PUFA and control groups with regard to appetite as measured by visual analogue scale, *ad libitum *food intake, resting energy expenditure as measured by indirect calorimetry, diurnal plasma leptin concentrations, or fasting ghrelin concentrations.

**Conclusion:**

Our results suggest that dietary n-3-PUFA do not play an important role in the regulation of food intake, energy expenditure, or body weight in humans.

## Introduction

Overweight and obesity are major public health concerns [1], and effective long-term treatments for these conditions are lacking [2]. Foods that reduce appetite or increase satiety or energy expenditure might be helpful in achieving and sustaining weight loss. Diets rich in n-3-PUFA are associated with lower plasma leptin concentrations than diets with a low n-3-PUFA content in healthy humans [3-5]. The fact that reduced leptin concentrations in these studies were not associated with increased hunger or decreased energy expenditure suggested that dietary n-3-PUFA might increase leptin sensitivity. Indeed, consumption of diets rich in n-3-PUFA has repeatedly been associated with reduced food intake, increased energy expenditure, and/or lower body weight and fat mass – all indicators of increased leptin 'action' – in rats [6,7], mice [8,9], and one short-term study in humans [10]. Remarkably, a lower body weight or fat mass was observed in studies that controlled calorie intake, such as in the study of Baillie et al. who pair-fed the fish oil fed mice to mice receiving a control diet low in n-3-PUFA [6], and in *ad libitum *feeding studies [7,8,10]. As well-controlled longer-term studies in humans were lacking, we conducted a randomized controlled 16-week trial in healthy overweight or moderately obese men and women to test the hypothesis that a diet rich in n-3-PUFA from both plant and marine sources would reduce body weight by decreasing appetite and *ad libitum *food intake and/or by increasing resting energy expenditure.

## Experimental methods

### Subjects

Subjects were 26 healthy overweight or mildly obese (body mass index 28–33 kg/m^2^) men and women. Subjects were weight stable [within 2.27 kg (5 pounds) in the past 6 months] and within 4.54 kg (10 pounds) of their lifetime maximum weight. Exclusion criteria included the use of tobacco or recreational drugs, alcohol abuse, a history of cardiovascular disease or diabetes mellitus, the presence of any chronic or psychiatric illness, restrictive eating behavior, pregnancy, and the intake of selective serotonin-reuptake inhibitors, lipid-lowering drugs, β-blockers, glucocorticoids, or anabolic steroids. All subjects underwent informed consent procedures. The study protocol was approved by the University of Washington Institutional Review Board (#03-9082-D05), and was in compliance with the Helsinki Declaration.

### Study design

In the first two weeks of the study, all subjects consumed a control diet. Caloric content of the diet was calculated on the basis of a 3-day diet record completed by the subjects, and the Mifflin formula [11] adjusted for an activity factor based on a modified Blair physical activity questionnaire [12] to maintain each subject's weight to within 1 kg of baseline weight. Subjects were instructed to consume all food provided, not to eat any additional food, and to take a portion of their calculated daily fat intake as capsules filled with high-oleic safflower oil. They were asked to keep a daily food record and write down all the foods they had eaten, estimate the percentage eaten, and any deviations from the diet protocol. On the morning of day 14 of the control diet, subjects were admitted to the University of Washington Clinical Research Center (CRC) after an overnight fast of at least 10 hours for clinic visit #1 (CRC 1).

Upon completion of CRC 1, subjects were randomized to continue consuming the control diet or to consume a diet enriched in n-3-PUFA, for another two weeks. The n-3-PUFA diet was identical to the control diet in all respects apart from the sources of dietary fat. A portion of the augmented n-3-PUFA intake was provided by switching the subjects from capsules containing high-oleic safflower oil to an identical number of capsules containing fish oil, with the balance of the n-3-PUFA provided in prepared foods [13]. The n-3-PUFA diet contained 1.4% of energy in the form of the long-chain n-3-PUFA eicosapentaenoic acid (20:5n-3), docosapentaenoic acid (22:5n-3), and docosahexaenoic acid (22:6n-3), and 2.2% of energy in the form of α-linolenic acid (18:3n-3) from plant oils, while the control diet provided 0% of energy in the form of marine n-3-PUFA and 0.5% of energy as α-linolenic acid. Subjects continued to pick up food, turn in food records, and were weighed twice weekly. The total caloric content of dispensed food items was identical to the amount that led to weight stability in the lead-in diet period ('isoenergetic'). The subjects were again instructed to consume all food and not to eat any other food. On day 14 of this period, subjects were again admitted to the CRC for visit 2.

After CRC 2, subjects continued to consume the diets to which they had been randomized for another 12 weeks but now freely chose the amount they wished to eat (*ad libitum*). In this phase, the caloric content of food provided to subjects was 115% of the amount they had received in the previous isoenergetic period. Subjects continued to weigh twice weekly, pick up food and return uneaten food items, allowing an accurate assessment of the calories consumed on each day of the study. During each food pick-up visit, subjects met with a dietitian for about 15 minutes to review the food records and the returned, uneaten foods. This meeting and the comparison of the food records and returned foods were implemented as they provided valuable information about the compliance of the subject. Specifically, meeting with the subject in person twice weekly for the 16-week dietary period created a rapport with the subject and helped us respond in a timely fashion to specific requests such as switching specific meals or foods. Further, any major or repeated deviations from the study protocol would have required a substantial investment of time and effort from the subject to make sure that the amounts of foods returned were always consistent with those recorded on the food diary. To further increase compliance, subjects were allowed to drink up to 30 alcoholic drinks, and to exchange up to 10 study meals for "meals out" or meals prepared at home during the first 10 weeks of the *ad libitum *diet period. We asked subjects to provide detailed information about alcoholic beverages and meals out on the daily food record. Specifically, we asked them to note the amount and type of alcoholic beverage consumed, and to provide recipes for meals prepared at home, including details such as the amounts and sources of dietary fat. If they had a meal at a restaurant, we asked them to provide the names of the restaurant and the dish they had chosen. A dietitian of the CRC Nutrition Research Kitchen would then call the restaurant to obtain more detailed information about the dish. Of note, 18 out of the 26 subjects who completed the study exchanged a study meal for a "meal out" on less than the allowed 10 occasions, suggesting an excellent compliance with the dietary regimen. On day 84 of this diet period, subjects were again admitted to the CRC for visit 3.

During each of the three admissions to the CRC, subjects were weighed immediately after the first morning voiding. Breakfast, lunch, dinner and a snack were served at 0800, 1200, 1730, and 2000, respectively, and subjects were asked to complete each meal within 30 minutes. An intravenous line was used to sample 5 ml of blood every half-hour between 0800 and 2100, and every hour between 2100 and 0800 the next day. Additional fasting blood samples were drawn at 0800 on day 2 of each CRC visit and plasma prepared from all samples was kept frozen at -70°C until analyses. The 38 diurnal plasma samples were frozen individually, for measurement of plasma leptin concentrations. During CRC visits 1 and 3, a whole-body dual-energy x-ray absorptiometry scan was performed using a GE Lunar Prodigy (General Electric Healthcare, Waukesha, Wisconsin, USA) scanner to assess body fat mass. At 0700 on day 2 of each CRC admission, we performed indirect calorimetry for 30 minutes to assess resting energy expenditure using a TrueOne 2400 metabolic cart (Parvo Medics, Sandy, Utah, USA). Throughout the study, subjects completed daily visual analogue scale (VAS) questionnaires to assess hunger and fullness. They were also asked to complete a modified Blair physical activity questionnaire [12] every 2–4 weeks. Additional details about subject characteristics and study design have been published previously [13].

### Laboratory methods

Plasma leptin and ghrelin were measured in duplicate by commercially available radioimmunoassays (Millipore, Billerica, Massachusetts, USA). Leptin was measured in all 38 timed diurnal samples drawn during each of the three clinic visits. Ghrelin was measured in fasting samples, and in pools of plasma created from the timed blood samples by combining 50 μL plasma from each blood sample drawn at 30-minute intervals and 100 μL plasma from each blood sample drawn at 60-minute intervals ('pool'-sample). Intra- and interassay CV were 2.4% and 3.6%, respectively, for leptin, and 4.3% and 5.3%, respectively, for ghrelin.

### Statistical analyses

Sample size calculation revealed that completing 36 subjects would provide 80% power to find a difference in body weight of 2.25 kg between the n-3-PUFA and control groups. The estimate for the variability in body weight change over the 12-week *ad libitum *diet period was derived from two previous controlled dietary trials conducted by our group that had employed an identical design [14,15]. We determined that we wanted to perform one interim analysis after 26 subjects had completed the study, which would allow us either to stop the study for effect or futility or to continue until the full 36 subjects had completed the study. We therefore designed this study as a sequential clinical trial, using the Power Family of Two-Sided, Inner Wedge Tests (chapter 5 in [16]). This test was chosen as it ensures specified α-error and power conditions [16].

Analyses were performed using SPSS, version 11.5 (SPSS Inc., Chicago, Illinois, USA). Changes in body weight, body fat mass, fasting leptin, the area under the leptin curve, and the leptin peak-nadir were compared by repeated measures analysis of variance (RM-ANOVA), with the three time points (CRC 1, CRC 2, and CRC 3) as the three levels of the within-subjects factor (time), and treatment (control vs. n-3-PUFA) as the between-subjects factor. *Post hoc*, we performed another RM-ANOVA, with a reduced number of the within-subjects factor by including either just CRC 1 and 2, or CRC 2 and 3. If the assumption of sphericity did not hold, degrees of freedom were adjusted according to Greenhouse & Geisser. Friedman tests separately for each group were performed if the residuals were not normally distributed, which was the case only for physical activity. The level of significance was set to p < 0.05.

## Results

**Figure 1:**
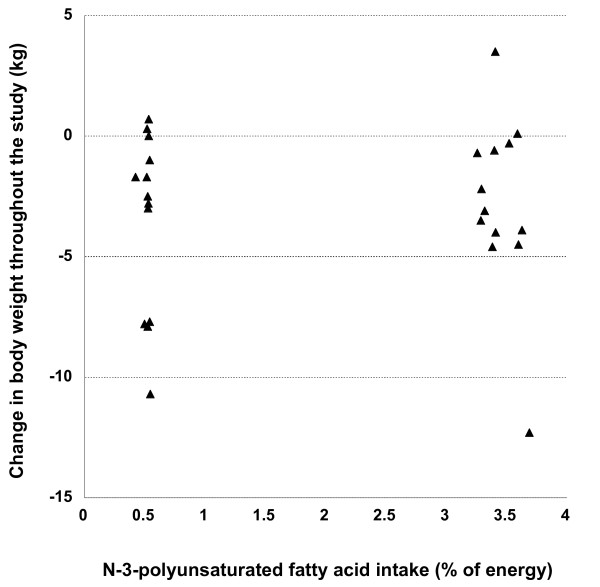
**Change in body weight in both groups, in relation to the average daily intake of n-3-polyunsaturated fatty acids**.

The one interim analysis that was performed according to the statistical protocol after 26 subjects had completed the study showed that both groups lost similar amounts of weight during the *ad libitum *phase [-3.5 (3.7) kg control vs. -2.8 (3.7) kg n-3-PUFA, mean (SD), Figure 1], which was significant overall [time effect in RM-ANOVA, F_(1,24) _= 13.425, p = 0.001], but not different between the two groups [time × group interaction, F_(1,24) _= 0.385, p = 0.541] [13]. We therefore stopped the study for futility, as determined in the formal stopping rules. Similarly, changes in body composition and particularly fat mass were similar between the two groups, as reported earlier [13].

**Figure 2:**
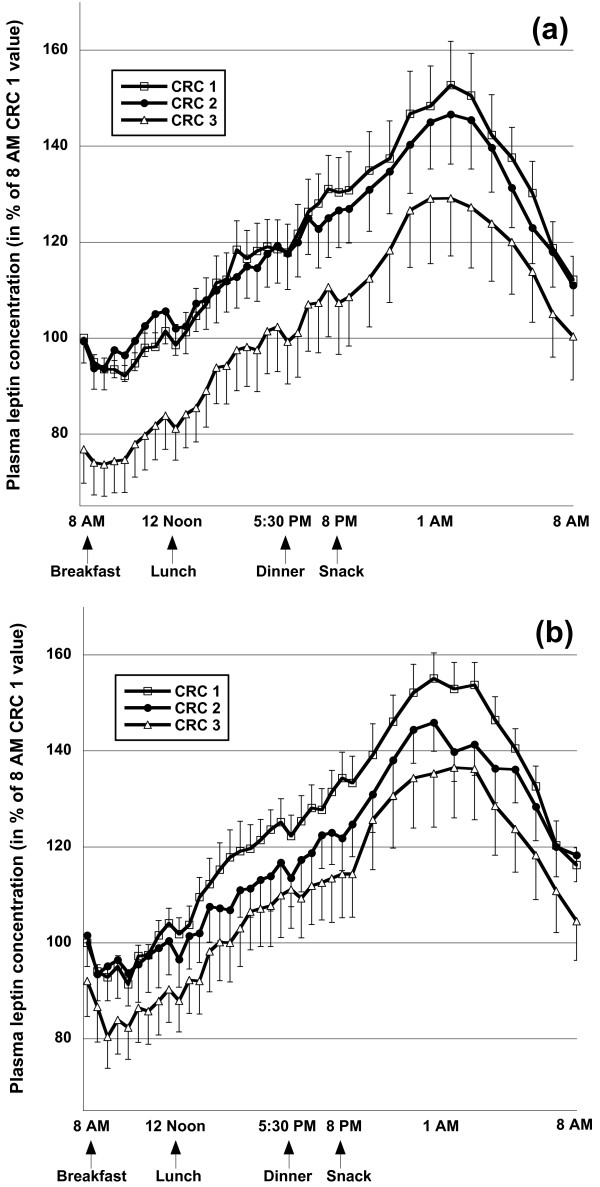
**Diurnal plasma leptin concentrations during clinical research center (CRC) visits 1, 2, and 3 in subjects consuming the control diet (a, n = 13) or the diet rich in n-3-polyunsaturated fatty acids (b, n = 13)**. Blood was drawn every 30' between 0800 and 2100, and every 60' thereafter. Meals were consumed at 0800, 1200, 1730, and 2000 immediately after the respective blood draws, and subjects were requested to complete the meal within 30'. Data are expressed in % of the CRC 1 0800 value, and represent means and standard errors of the means.

The area-under-the diurnal plasma leptin curve did not change between CRC 1 and CRC 2 in either group [time: F_(1,24) _= 1.927, p = 0.178; time × group: F_(1,24) _= 0.594, p = 0.449 in RM-ANOVA], but decreased when subjects lost weight during the *ad libitum *period by 450 ± 896 arbitrary units (AU) in the control group, and by 350 ± 705 AU in the n-3-PUFA group [time: F_(1,24) _= 6.394, p = 0.018, time × group: F_(1,24) _= 0.100, p = 0.754 in RM-ANOVA; Figure 2]. Fasting ghrelin concentrations in plasma increased moderately by ~7% in both groups when subjects lost weight during the *ad libitum *period, but were not different between the groups (Table 1). Changes in ghrelin concentrations in the 'pool'-sample reflecting the average ghrelin concentrations throughout each of the 24-hour clinic visits were similar to those observed in fasting plasma (Table 1).

**Table 1:** Plasma ghrelin concentrations throughout the study

				RM-ANOVA *	
	CRC 1 †	CRC 2	CRC 3	Time	Time × Group
				
Fasting plasma ghrelin (pg/mL):					
Control group (n = 13)	766 (118)	780 (127)	820 (139)	F_(2,48) _= 4.058;	F_(2,48) _= 0.071;
n-3-PUFA group (n = 13)	687 (189)	708 (244)	734 (241)	p = 0.024	p = 0.931
Plasma ghrelin in 'pool'-sample (pg/mL)					
Control group (n = 13)	760 (148)	770 (116)	800 (159)	F_(2,48) _= 4,799;	F_(2,48) _= 0.300;
n-3-PUFA group (n = 13)	687 (211)	706 (249)	718 (235)	p = 0.013	p = 0.742

All data are means (SD). * RM-ANOVA, repeated measures analysis of variance. † CRC, Clinical Research Center.

No changes were observed in hunger and fullness ratings as assessed by VAS throughout the study (data not shown). Compared to the isoenergetic periods, food intake tended to be lower in the *ad libitum *period in both groups [-0.74 ± 1.47 MJ/d control vs. -0.39 ± 1.61 MJ/d n-3-PUFA, time effect in RM-ANOVA: F_(1.100,26.409) _= 2.994, p = 0.092], but without any difference between the groups [time × group interaction: F_(1.100,26.409) _= 0.395, p = 0.555; Table 2]. Similarly, resting energy expenditure as the major component of daily energy expenditure did not change throughout the study, and was not different between the two groups [time: F_(2,46) _= 0.313, p = 0.733; time × group: F_(2,46) _= 0.703, p = 0.500 in RM-ANOVA]. Lastly, physical activity remained constant in both groups throughout the study [χ^2^_(5) _= 6.156, p = 0.291 for control and χ^2^_(5) _= 7.313, p = 0.198 for n-3-PUFA group, Friedman tests; Table 2].

**Table 2:** Calorie intake, physical activity, and resting energy expenditure throughout the study

	Lead-in period (2 weeks)	Isoenergetic period (2 weeks)	*Ad libitum *period (12 weeks)	Friedman tests	
	
Physical activity (MET-h/d)*:					
Control group (n = 13)	38.2 (19.9)	38.6 (22.7)	37.4 (21.7)	χ^2^_(5) _= 6.156, p = 0.291	
n-3-PUFA3 group (n = 13)	54.8 (29.9)	62.0 (29.9)	57.5 (29.6)	χ^2^_(5) _= 7.313, p = 0.198	
				RM-ANOVA†	
				Time	Time × Group
				
Calorie intake (MJ/d):					
Control group (n = 13)	10.9 (1.5)	10.9 (1.3)	10.2 (1.9)	F_(1.1,26.4) _= 2.994,	F_(1.1,26.4) _= 0.703,
n-3-PUFA group (n = 13)‡	11.0 (2.2)	11.1 (2.0)	10.7 (2.5)	p = 0.092	p = 0.555
	CRC 1 §	CRC 2	CRC 3		
			
Resting energy expenditure (MJ/d):					
Control group (n = 12)	6.74 (0.89)	6.41 (1.10)	6.41 (0.79)	F_(2,46) _= 0.313,	F_(2,46) _= 0.703,
n-3-PUFA group (n = 13)	6.53 (1.23)	6.59 (1.51)	6.57 (1.39)	p = 0.733	p = 0.500
Resting energy expenditure (kJ/d/kg fat free body mass ||):					
Control group (n = 12)	134 (15)	-	132 (13)	F_(1,23) _= 0.002,	F_(1,23) _= 0.315,
n-3-PUFA group (n = 13)	133 (21)	-	135 (14)	p = 0.961	p = 0.580
Respiratory quotient:					
Control group (n = 12)	0.86 (0.05)	0.87 (0.05)	0.86 (0.05)	F_(2,46) _= 0.096,	F_(2,46) _= 1.091,
n-3-PUFA group (n = 13)	0.85 (0.03)	0.84 (0.05)	0.85 (0.05)	p = 0.908	p = 0.344

All data are means (SD). * MET-h, metabolic equivalent task-hours. † RM-ANOVA, repeated-measures analysis of variance. ‡ PUFA, polyunsaturated fatty acids. §CRC, Clinical Research Center. || Fat-free body mass data from dual-energy x-ray absorptiometry-scans were available only for CRC visits 1 and 3.

## Discussion

Our well-controlled 16-week study showed that increasing dietary n-3-PUFA content to 3.6% of total energy did not have an effect on plasma leptin levels, body weight or fat mass in overweight or moderately obese, healthy men and women. We designed the study as a sequential clinical trial, using the power family of two-sided, inner wedge tests (chapter 5 in [16]). This test was chosen as it ensures specified α-error and power conditions [16], indicating we had sufficient power to confidently accept the null hypothesis of no difference between the two groups at this point.

We decided not to administer more than 3.6% of energy in the form of n-3-PUFA, as higher intakes are not realistic over an extended period of time in a free-living population. Endpoints affected by leptin signaling such as resting energy expenditure, appetite, or *ad libitum *food intake were not different between the n-3-PUFA and control diets, suggesting that n-3-PUFA do not increase leptin sensitivity as hypothesized. Our findings are consistent with those of Krebs and colleagues [17] who studied cardiovascular endpoints in 93 overweight women randomized to a weight loss diet with or without n-3-PUFA (fish oil), or to a control diet. Weight loss over 12 and 24 weeks was independent of the n-3-PUFA content of the diets.

These results are in contrast, however, to several studies in rodents [6-9], and one small short-term study in humans [10]. Couet et al. fed normal weight subjects control diets for 3 weeks, followed by diets rich in n-3-PUFA for another 3 weeks [10]. While weight loss in these two periods was similar, the subjects lost more fat mass when they consumed the n-3-PUFA-rich diet (-0.88 ± 0.16 vs. -0.30 ± 0.34 kg, p = 0.02). Based on our results and those of Krebs and colleagues, it seems possible that the findings by Couet et al. were due to chance based on their small sample size and short study duration, or due to differences between the study diets other than n-3-PUFA.

Strengths of our study include the fact that all foods were provided for a period of 16 weeks, and that the quality and quantity of food consumed was assessed for each day of the study. The n-3-PUFA and control diets were designed to be identical except for the fatty acid composition. As all subjects were seen at least twice weekly by study staff, and were encouraged to share with us any deviation from the study protocol, we feel confident in our assessment of their actual intake. Nevertheless, the fact that subjects could have been non-compliant is a potential limitation of this study.

We conclude that dietary n-3-PUFA do not have a significant effect on plasma leptin and ghrelin concentrations, appetite, *ad libitum *food intake, resting energy expenditure, or body weight and fat mass in overweight or moderately obese men and women.

## Competing interests

The authors declare that they have no competing interests.

## Authors' contributions

MK and DSW planned and designed the study, and were responsible for all aspects of the project including subject recruitment, data collection and management, laboratory analyses, statistical analyses and interpretation of the data, and writing of the first draft of the manuscript. HSC and CCM were responsible for all diet-related aspects of this study including recipe development, diet composition calculations, diet preparation, and weigh-back of returned foods. PYY was involved in blood sample collections and processing, and was responsible for laboratory measurements of diurnal leptin and fasting ghrelin plasma concentrations. All authors were involved in the interpretation of the study results, and the writing of the manuscript. All authors read and approved the final version of the manuscript.
